# Beyond the Immune Suppression: The Immunotherapy in Prostate Cancer

**DOI:** 10.1155/2015/794968

**Published:** 2015-06-16

**Authors:** Ida Silvestri, Susanna Cattarino, Anna Maria Aglianò, Giulia Collalti, Alessandro Sciarra

**Affiliations:** ^1^Department of Molecular Medicine, Sapienza University of Rome, 00161 Rome, Italy; ^2^Department of Urology, Sapienza University of Rome, 00161 Rome, Italy; ^3^Department of “Medicina dei Sistemi” Rheumatology, Allergology and Clinical Immunology, University of Rome “Tor Vergata”, 00133 Rome, Italy

## Abstract

Prostate cancer (PCa) is the second most common cancer in men. As well in many other human cancers, inflammation and immune suppression have an important role in their development. We briefly describe the host components that interact with the tumor to generate an immune suppressive environment involved in PCa promotion and progression. Different tools provide to overcome the mechanisms of immunosuppression including vaccines and immune checkpoint blockades. With regard to this, we report results of most recent clinical trials investigating immunotherapy in metastatic PCa (Sipuleucel-T, ipilimumab, tasquinimod, Prostvac-VF, and GVAX) and provide possible future perspectives combining the immunotherapy to the traditional therapies.

## 1. Introduction

Prostate cancer (PCa) is the second most common cancer in men. Although an estimated 15% of the cancers occurred in men in most developed countries, incidence rates are also relatively high in certain less developed regions. It represents the fifth leading cause of death from cancer in men [1]. Therefore screening and management of early prostate cancer are critical medical challenges. Even though the precise aetiology is not completely defined, both hereditary and environmental factors are important in the development of PCa. Human and animal studies suggest that the inflammation and the elusion of immune destruction can have an important role in PCa as well in the development of many other human cancers [2]. Immune evasion is now recognized as a hallmark feature of cancer [3].

**Figure 1:**
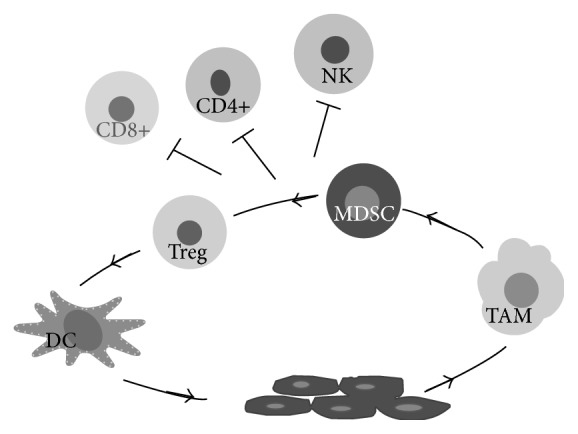
MDSCs produce high amount of IL-10 and drive polarized macrophage M2 (TAM) and active Tregs. MDSCs, TAMs, and Treg produce a cytokine subsets that interfere with DCs differentiation and enhance the suppressive phenotype of each cell type and inhibit CD4^+^, CD8^+^, and NK, and promove tumor progression. Tumor cells produce: PGE, COX, IL-6, VEGF, and other factors that recruit MDSCs, TAMS, and Tregs, induce defective DCs, and induce an immune suppressive microenvironment.

Generally the inflammatory process restores the homeostasis but especially the chronic inflammation can produce a microenvironment that supports cancer initiation and progression [4, 5]. In addition to this extrinsic pathway, genetic alterations leading to cancer can also stimulate the inflammatory process, thus contributing to the establishment of a microenvironment favorable to tumor progression (Figure 1) [4]. Recent studies have demonstrated that the interaction between immune system inflammation and cancer is very complex and still far from fully understood, ranging from positive local effects, such as cytotoxicity mediated by T-cells to tumor progression until the destructive systemic effects such as cachexia [6].

In cancer patients immunity system is often altered with an excess of inhibitory functions induced by regulatory T cells (Treg) or myeloid-derived suppressor cells (MDSC) and by secretion of the immunosuppressive cytokines, tumor growth factor (TGF)-*β*, and interleukin (IL)-10. The manipulation of the immune system is also one of new promising therapies for cancer treatment, as detected in many different tumors (colon, breast, melanoma, and prostate) but until now only rarely established durable effects have been demonstrated [7]. Several ongoing trials have the purpose to identify new therapies that interfere with synergic activity of immunosuppressive environment and restore immune competence.

The aim of this review is to describe some of the agents that can activate different pathways involved in PCa promotion and progression, with particular interest to those leukocytes that inhibit immunity response to cancer. We also specify some of the potential strategies aimed to alter cancer associated inflammation-immunity that are focused on the components of the tumor microenvironment.

## 2. Immunosystem in the Prostate Gland

An immune response in the prostate has been reported, and it is primarily cell-mediated [8]. The greatest concentration is in the stroma with a small but significant number of intraepithelial cells. The lymphocytes are chiefly T cells (CD45RO^+^) in both stromal and intraepithelial compartments. Stromal T cells are mainly CD4^+^ helper/inducer cells, whereas intraepithelial cells are CD8^+^ cytotoxic/suppressor. The abundance of CD8^+^ suggests that cytotoxic T cells are the first line of defense against luminal foreign agents. CD4^+^ T cells can have different fates and are classified according to their cytokine profile: T helper (Th)-1 and T helper (Th)-2. Th1 express T-bet and produce interferon (IFN)-*γ*; Th2 express Gata-3 and produce IL-4 [9]. Tregs are CD4^+^ lineage with essential immunosuppressive functions that often express transcription factor like Forkhead box P3 (FoxP3^+^). Other T cells selectively produce IL-17 and the transcription factor ROR*γ*t (Th17), and finally newer T cells are identified and they are defined based on their cytokine production: Th9 and Th22 cells.

As helper cells can change their phenotype, it has become important to determine which T cells are more present in inflammatory lesions of prostate from BPH until carcinoma. In fact, an immune response is stimulated in PCa, as shown also by histological data revealing the presence of CD4^+^ T cells, CD8^+^ T cells, natural killer (NK) cells, dendritic cells (DC), and macrophages within tumors. Furthermore, it has been reported that a dense infiltration of lymphocytes is correlated with longer patient survival and that high grade prostatic adenocarcinomas have significantly less infiltration of T cells and macrophages as compared to benign nodular prostatic hyperplasia [10–13], suggesting that tumor progression may be associated with alterations in cell-mediated immune responses. On the contrary, [14] an increased inflammatory cell infiltrate within the tumor is associated with an increased risk of tumor recurrence. In some reports the presence of CD4^+^ T cells infiltrate is related with poor cancer survival in patients with PCa, probably due to an increase of Tregs. So far it seems the presence of tumor associated macrophage (TAM) and Tregs correlate with a poorer prognosis [4, 12]. A cross-talk between these cells could promote synergy and amplify the immune suppressive effects of individual cell population [15].

### 2.1. Regulatory T Cells

CD4^+^CD25^+^ Tregs represent the major Treg population in the immune system [16] and are essential to maintain peripheral self-tolerance and avoid autoimmunity. They are also responsible of limiting tissue damage during ongoing and resolving immune responses [17, 18]. Expression of Foxp3^+^ generally identifies natural, thymus derived Treg cells (nTregs) and may or may not be expressed in inducible Tregs (iTregs) [19–21]. Foxp3^+^Tregs were detected in the peripheral blood and tumor tissue in many cancer patients suggesting their contribution to the reduction of the antitumor immune response [22]. The recruitment of Tregs (natural or induced) into tumors likely involves complex, multistep processes not yet completely defined [22, 23].

Tregs generally contribute to decreasing immunity during tumor development and progression, leading to poor outcomes in cancer patients [24, 25]. Likewise a relative enrichment of Tregs has been detected in prostate tissue and from peripheral blood of PCa patients compared to normal donors [26]. A significant association has been shown between the number of Tregs and poor prognosis in PCa [27, 28]. Moreover Tregs level decreases after androgen ablation and is elevated in the peripheral blood of patients with metastatic castration-resistant prostate cancer (mCRPC) [26, 28–30].

The mechanisms of suppression mediated by Tregs include cytotoxic T-lymphocytes-associated protein (CTLA)-4, programmed death-ligand (PD-L)-1, lymphocyte-activation gene (LAG)-3, neuropilin (Nrp)-1, and CD39/73 expression [31].

### 2.2. Myeloid-Derived Suppressor Cells

MDSCs are elevated at the tumor site, as well as in the peripheral blood of cancer patients and a correlation between tumor-MDSCs and patients survival has been described [32]. MDSCs are a heterogeneous cell population characterized by the ability to suppress T cells and NK cells functions. They consist of myeloid progenitor cells and immature myeloid cells (IMC). IMCs with a phenotype as MDSCs are continually generated in the bone marrow of healthy individuals and differentiate into mature myeloid cells without causing detectable immunosuppression [33]. Some pathological conditions, such as acute or chronic infections, trauma or sepsis, and cancer, prevent the differentiation and MDSCs exhibiting immunosuppressive functions derive [34, 35]. Until now two main MDSC populations have been characterized primarily in mice: polymorphonuclear and monocytic MDSC. These cells share some characteristics but have also many different markers that complicate their studies and lead to controversial results. Nevertheless, there is a growing consensus to define human MDSCs as CD11b^+^CD33^+^HLA-DR^low/−^Lin^−^. Within this population, the CD14^+^CD15^low/−^ MDSCs share characteristics with murine M-MDSCs, while CD14^−^CD15^+^MDSCs resemble murine G-MDSCs [36]. Several different factors, including cyclooxygenase (COX)-2, prostaglandins (PGE), stem-cell factor (SCF), Macrophage-Colony Stimulating Factor (M-CSF), IL-6, granulocyte/macrophage CSF (GM-CSF), and vascular endothelial growth factor (VEGF), induce expansion and activation of MDSCs. These factors are also produced by tumor cells and promote the expansion of MDSCs through the stimulation of myelopoiesis and the inhibition of the differentiation in mature myeloid cells. In addition other signals are necessary to MDSCs activation, including IFN-*γ*, ligands for Toll-like receptors (TLRs), IL-4, IL-13, and TGF-*β*, produced mainly by activated T cells and tumor stromal cells after induction by different bacterial and viral products, or as a result of tumor-cell death [33].

The immunosuppressive activities of MDSCs are mediated by a variety of mechanisms. One mechanism consists in the depletion of essential nutrients, especially L-Arg, metabolized by arginase 1 and iNOS highly expressed in MDSCs. The depletion of Arg inhibits T-cell proliferation by decreasing their expression of CD3 *ζ*-chain and preventing upregulation of the expression of the cell cycle regulator, cyclin D3 [33, 37, 38]. Another mechanism is the generation of oxidative stress caused by the production of reactive oxygen species (ROS) and reactive nitrogen species by MDSCs, able to produce several molecular blocks in T cells, ranging from the loss of TCR *ζ*-chain expression and interference with IL-2 receptor signaling [33, 39]. MDSCs also disrupt T cell migration to lymph nodes by releasing ADAM 17 which down regulates L-selectin and prevents the homing receptor on T cells [40]. At last, MDSCs promote the recruitment and the expansion of Tregs by the production of IL-10, TGF-*α*, IFN-*γ*, and by CD40–CD40L interactions [41, 42]. In addition MDSCs enhance tumor growth by promoting angiogenesis [43], inducing tumor invasion and metastasis, and activating the protective pathways of tumor cells from chemotherapy-induced apoptosis [15, 33, 42].

Monocytic MDSCs have been detected elevated in the peripheral blood of patients with PCa and the level of MDSCs correlated with other negative prognostic factors for metastatic PCa, such as lactate dehydrogenase, alkaline phosphatase, PSA, and anemia [44]. It has also been described that CD14^+^HLA-DR^low/−^ monocytes isolated from PCa patients expressed high level of IL-10, inhibited autologous T cell proliferation more effectively than (CD14^+^HLA-DR^+^) monocytes from healthy individuals, and were defective in their ability to differentiate into phenotypically mature DCs [45].

### 2.3. Macrophages

Macrophages play a basic role that promote host survival by regulating adaptive immunity, inducing wound healing and eliminating infectious agents [46]. Their precursor cells, monocytes, after extravasation into target tissues differentiate to mature macrophages and polarize in response to microenvironment. Each polarized macrophage displays a differential expression profile of cytokines, enzymes, and cell-surface markers and they have been classified into two subsets. The classical M1, activated by IFN-*γ* and lipopolysaccharide (LPS), are characterized by their high expression of IL-12 and low expression of IL-10; the alternative M2, that are activated by IL-4, IL-13, IL-10, and glucocorticoid hormones, produce high levels of IL-10 and low levels of IL-12 [47, 48]. The role of macrophage in tumor development has been controversial. Even though macrophage surveillance mechanisms are essential for preventing the growth of transformed cells, activated macrophages contribute to early development of neoplasm through the free radicals production. Furthermore tumor microenvironment strongly polarizes macrophages towards a M2-like phenotype, the so-called TAMs, which facilitate tumor progression via both immunological and nonimmunological mechanisms. In fact in tumor microenvironment, molecules such as chemokines (CCL-2), cytokines (VEGF and M-CSF), and hypoxia promote monocytes recruitment as well as macrophages survival. TAMs expression correlates with tumor growth [49]. Often the same factors inhibit the differentiation of DCs. In turn, the recruited macrophages provide a transcriptional program, activated through Nuclear factor (NF-kB) and hypoxia-inducible transcription factors (HIF)-1, which support tumor progression and metastasis [4, 50, 51].

For many years a strict correlation between an increased number of macrophages and a poor prognosis has been described for many different tumors. TAMs are also a significant component of the inflammatory infiltrates in PCa. The detection of high density of M2 in both epithelial and stromal compartments was statistically associated to poorer prognosis [52, 53]. Moreover increased TAMs levels in biopsy are predictive of worse recurrence free survival in men treated with primary androgen deprivation therapy. An inverse correlation between total macrophage density and time to recurrence has also been reported from different analysis [54, 55].

### 2.4. Dendritic Cells

DCs are professional antigen-presenting cells (APC), which are critical to initiate innate and adaptive immune responses against pathogens and tumor cells, and because these cells orchestrate a large repertoire in T cell activation representing also a good tool for DCs-based cancer vaccination strategies [56].

DCs are terminal differentiated myeloid cells that are specialized in antigen processing and presentation. These cells differentiate in the bone marrow from various progenitors. In human, monocytes represent the major precursors of DCs. The differentiation leads to two major subsets of DCs, conventional DCs (cDCs), and plasmacytoid DCs (pDCs). They show different morphologies, markers, and functions. Pathogen-associated molecular patterns (PAMPs) and damage-associated molecular patterns (DAMPs) induce different pathway of differentiation in DCs [57]. The different pathways of differentiation define the fate of DCs and their interaction with lymphocytes. In fact activated DCs produce a different setting of costimulatory molecules and cytokines inducing such contrasting states as immunity and tolerance. DCs after capturing and processing antigens present them to T cells through MHC and, by controlling Th1, mediate a resistance to intracellular microbes, by Th2 a defense to helminthes, by Th17 through IL-17 organize phagocytes at body surface to resist extracellular bacilli. Alternatively, DCs induce Tregs and cause tolerance. Maturing DCs also express more IL-15 and activate inflammation and NK [57].

DCs maturation is induced by tumor derived molecules, such as heat shock proteins (HPS) and high mobility-group box- (HMGB-) 1 protein, as well as proinflammatory cytokines produced by various tumor-infiltrating immune cells. Matured DCs have different tumoricidal activities often mediate by IFN production. DCs activate T cells and NK cells, both these cells have cytotoxic activity against tumors. DCs induce apoptosis and antiangiogenesis pathways via signaling through IFN [58]. Alternatively tumor may perturb this process by inducing the accumulation of immature DCs [33, 59]. The contact tumor-DCs or tumor-derived factors may affect DCs maturation and function. It has been demonstrated that tumor induces apoptosis or alters differentiation of DC as well as accumulation of immature cells with inhibitory function could impair immune responses [59, 60]. Defective DCs function has been found in many patients with a variety of cancers [61].

Some authors have detected in prostate carcinoma a significant correlation between low numbers of CD1a^+^ cells (characterized DCs) and a high Gleason score, by contrast; DCs have been found elevated in low risk cancer [62]. Patients that suffer from metastatic PCa showed fewer circulating myeloid DCs than their age-matched controls [63]. These results indicate that in PCa patients monocytes do not develop into myeloid DCs as efficiently as they do in healthy individuals. This idea is also supported by observations that serum from PCa patients inhibited monocyte differentiation into DCs and that the degree of inhibition correlated with higher PSA levels [64].

## 3. Immunotherapy in Prostate Cancer: Clinical Data

Cancer immunotherapy has recently been introduced into the therapeutic field of metastatic PCa and mCPRC. The goal of immunotherapy is to harness the capabilities of immune system to effectively recognize and kill transformed cells whilst sparing healthy tissues [65, 66]. Over the past decade, strong evidences that PCa is immunogenic have emerged, which showed the rationale for using immune-based therapies for the treatment of metastatic PCa. This is confirmed by the support of the presence of several tumor associated antigens in the prostate; which include the PSA, prostatic acid phosphatase (PAP), prostate specific membrane antigen (PSMA), prostate stem cell antigen (PSCA), mucin-1 (MUC-1), and the cancer testis antigen NY-ESO-1. As already mentioned, histological data revealed the presence of CD4^+^, CD8^+^ T cells, NK cells, DCs, and macrophages within tumors [7, 11]. Early studies reported that high grade PCa have significantly less infiltration of T cells, suggesting that tumor progression could be associated with defects in cell-mediated immune responses. A high prevalence of Tregs within tumors is associated with more lethal PCa, suggesting that therapeutic blockade of these cells may induce beneficial clinical response. Another recent observation consists in a reduced infiltration of CD68^+^ macrophages that is associated with lymph node positivity and higher clinical stage. Increased NK infiltrate within tumors was also found to be associated with a lower risk of progression providing evidence that these innate immune cells may have a protective role against PCa. Four of the current immunologic therapeutic approaches with particular relevance to mCPRC are discussed in more detail in this section of the review (Table 1).

*Sipuleucel-T* is an autologous active cellular immune-therapy product that stimulates a T-cell immune response against cancer cells [65]. It is the first documented immunotherapy to prolong survival in mCRPC demonstrated in a phase III trial [67]. Autologous peripheral blood mononuclear cells (PBMCs) of patient are incubated* ex vivo* for 36/48 hours with a fusion protein (PA2024) of PAP and GM-CSF [68]. After about 40 hours, the fusion protein is washed out and the product is reinfused into the patient. This product contains at least 5 × 10^7^ autologous activated CD54^+^ DCs and a variable number of T cells, B cells, NK cells, and others [74]. Sipuleucel-T immunotherapy targets cells positive for PAP, a secreted glycoprotein enzyme that is expressed in 95% of prostate tissue and PCa [75]. Phase I/II clinical trials have shown that Sipuleucel-T is well-tolerated and the patients developed an appreciable antigen specific T-cell responses and antibodies against the fusion protein after the treatment [69, 70]. Actually three phase III clinical trials have been completed and showed promising findings of this DCs based vaccine. The two first studies compared patients with asymptomatic mCRPC assigned to placebo or Sipuleucel-T. There was no difference in time to progression but there was a significant increase of overall median survival (25.9 months versus 21.4 and 19.0 months versus 15.7) [76, 77]. A third phase III clinical trial known as Immunotherapy for Prostate Adenocarcinoma Treatment (IMPACT) trial showed a 4.1 months improvement in median OS and at 36-month interval the survival rate was 31.7% for treated patients compared to 23.0% for cases treated with placebo [67, 78].

*Ipilimumab* is a fully human IgG1 monoclonal antibody that Bind to and blocks the activity of CTLA-4. CTLA-4 has been shown to be potent negative T cells responses and is upregulated following T-cell stimulation to attenuate the response. CTLA-4 is also constitutively expressed on Tregs and mediates their immune suppressive effects [71]. Ipilimumab was approved by the FDA on 2011 for the treatment of advanced melanoma and is currently being trialled for the treatment of nonsmall cell lung cancer, metastatic renal cell cancer, and ovarian cancer. Regarding PCa, preclinical studies that combine ipilimumab with standard anticancer therapies are giving encouraging results. Synergic antitumor activity between radiotherapy and CTLA-4 blockade has emerged in a phase I, II study. In this study ipilimumab given alone in a dose escalation or in addition to a single fraction of radiation each day before starting the treatment, resulted in some very significant PSA declines [72]. There are currently phase III trials that are evaluating the effect of ipilimumab in patients with metastases who received or not docetaxel. A primary analysis showed no significant difference between the ipilimumab group and the placebo group in terms of overall survival. However, ipilimumab seems to be associated with better survival than placebo [73].

*Tasquinimod,* a quinoline-3-carboxamide analog, is in clinical development for treatment of prostate cancer and other solid tumors. In a placebo-controlled, phase II randomized trial, tasquinimod doubled the median progression-free survival (PFS) period and prolonged survival of patients with metastatic CRPC [79, 80]. A phase III clinical trial to test the effect of tasquinimod in the same patients population is ongoing (NCT01234311). Tasquinimod has been shown to inhibit prostate cancer growth and metastasis in animal models [81, 82]. Results from these studies have suggested that the antiangiogenic property of this molecule may be responsible for its antitumor activity, since tumor growth inhibition was associated with reduced microvasculature density, increased expression and secretion of the angiogenesis inhibitor thrombospondin-1 (TSP-1), and downregulation of VEGF and HIF-1*α* [83]. More recent data have suggested that tasquinimod may affect HIF by interfering with histone deacetylase 4 (HDAC 4) [84]. However, in an orthotopic, metastatic prostate cancer model, tasquinimod reduced the metastatic rate without affecting microvessel density in the primary tumor. Therefore, mechanisms other than impairing angiogenesis may play an important role in the antitumor and antimetastasis activities of tasquinimod. At this regard, S100A9 has been identified as a potential target of tasquinimod. S100A9 interacts with proinflammatory receptors: TLR4 and receptor of advanced glycation end products (RAGE). These receptors are expressed on MDSC, macrophages, DCs, and endothelial cells. S100A9 regulates the accumulation of MDSCs and inhibits DCs differentiation leading to immune response suppression [85].

*Viral vectors* are attractive for use in cancer immunotherapies as they can mimic natural infection and lead to the induction of immune response against the tumor antigen that they encode.

*Prostvac-VF* (viral-based vaccine) is a recombinant viral vaccine currently being trialled as an immunotherapy for PCa. Prostat-VF (TRICOM or PSA TRICOM) is based on a combination of two viral particles, vaccinia which is a potent immunologic priming agent, followed by fowlpox which is minimally or noncross reactive with vaccinia that is used as a boosting agent. Both recombinant viruses are engineered to encode the entire PSA gene with a modified agonist epitope and three costimulatory proteins B7-1 (facilitates T cell activation), lymphocyte function/associated antigen 3 (LFA-3; CD58), and intercellular adhesion molecule-1 (ICAM-1; CD54). The rationale behind this approach is that the virus will directly infect the APCs or somatic cells at the site of injection, leading to cell death and subsequent uptake of cellular debris containing PSA by the APCs [86]. The transduced APCs or antigen-loaded APCs upon interaction with CD4^+^ and CD8^+^T cells will effectively promote the T-cell mediated immune responses that destroy PSA expressing cells. Prostvac-VF/TRICOM was evaluated in a randomized phase II clinical trial in men with mCPRC. Comparing men who received Prostvac-VF and GM-CSF and men received empty vector plus placebo, this study showed positive results in median OS with a difference of eight months between treated groups. Medians OS in the control group was 16.6 versus 25.1 months in PROSTAVAC group [87]. There is also currently a global phase III trial that included 1200 men with mCRPC treated with PROSTAVAC or placebo that will determine the overall survival.

*GVAX,* granulocyte-macrophage colony-stimulating factor tumor cell vaccine, represents the whole-cell based immunotherapy. Whole autologous or allogeneic tumor cells as source of immunogens are genetically modified to express GM-CSF. GM-CSF enhances immune responses through the recruitment and activation of DCs at the injection site, necessary to process and present antigens, a critical step in the induction of an optimal immune response to any immunotherapy [88]. Because the small number of cells that can be obtained from surgically removed tumors limits autologous approach, GVAX for PCa is composed of two human prostate cell lines, LANCaP (androgen sensitive derived from a lymph node metastasis) and PC3 (androgen insensitive derived from bone metastasis) as antigens source, transfected with GM-CSF, and then irradiated for safety [89, 90]. Phase I/II trials were performed: patients with hormone-refractory prostate cancer (HRPC), chemotherapy-naive, received an intradermal priming vaccination with GVAX-PCa (5 × 10^8^ cells, half quantity of each cell line) followed by 12 weekly boost for 6 months [89] or ranged doses (1 × 10^8^ cells to 5 × 10^8^ cells) [90]. This immunotherapy resulted well tolerated and immunogenic for many of metastatic HRPC patients in terms of dose and time treatment with an encouraging OS rates. These data supported to initiation of two phase III trials to confirm the survival benefits. The first phase III study, Vaccine Immunotherapy with Allogeneic Prostate Cancer Cell Lines (VITAL)-1, was a phase III trial designed to compare GVAX to docetaxel plus prednisone in asymptomatic CRPC [91, 92]. VITAL-2 was conducted in symptomatic CRPC [91, 92]. The VITAL-2 study was terminated early due to increased deaths in the vaccine arm. Not long after, the VITAL-1 study was terminated based on a futility analysis of less than a 30% chance of meeting its end point [91].

The failure of GVAX immunotherapy to demonstrate clinical benefit in PCa has urged some considerations. The critical points are the lack of placebo arm and dose levels and the timing of chemotherapy was not conducted. Moreover the effects of immunotherapy may need a longer time than conventional therapy; other biological markers may be necessary to determine the effect of immunotherapy and finally the development of immunosuppression especially in metastatic disease that may require a different approach [91]. Emerging data suggest that this effect may be at least partially obviated by combining immunotherapy with immune checkpoint antagonist or immune agonist [91]. In this regard, a combined treatment with GVAX and ipilimumab in patients with metastatic CRPC was trialled. The tolerable dose and the safety profile resulted in a phase I study warrant further research [92]. In addition results from a trial including mCRPC patients treated with fixed initial doses of ipilimumab and PSA-Tricom vaccine have shown a raise of the median overall survival [93].

## 4. Conclusions

Immunotherapies have gained momentum in cancer therapeutics following the recent approvals of drugs for the treatment of prostate cancer and melanoma. Immunotherapy has the potential to mount an ongoing, dynamic immune response that can kill tumor cells for an extended time after the conventional therapy has been administered. Despite these clinical advances, further studies are still necessary to increase the understanding of the responses to these types of therapy and of the optimal management of different subset of patients. The most promising immune-based treatments are monoclonal antibodies that act as checkpoint inhibitors (e.g., ipilimumab and nivolumab), adoptive cell therapy (e.g., T cells expressing chimeric antigen receptors), and vaccines (e.g., Sipuleucel-T).

It is reasonable that a single immune therapeutic agent is unlikely to be clinically effective especially in metastatic patients and combing vaccines with immune check-point inhibitors can reorganize the immunological network to mount an immune response against the prostate cancer antigens.

Many trials are also ongoing to define the effects of immune therapy with established treatments: androgen deprivation therapy (ADT) and chemotherapy (CT) or radiotherapy (RT) [94]. As mentioned above, studies about possible combine immunotherapies are ongoing to better establish the safety and toxicity other than the efficacy of such treatments. In our opinion, identifying doses and timing and the sequences of combined treatments are crucial to gain a synergic effect.

## Figures and Tables

**Table 1:** Current immunologic therapeutic approaches in PCa.

Therapy	Molecule	Mechanism of action	Clinical trials [Ref.]
Sipuleucel-T (Provenge)	Autologous cellular immune-therapy	Stimulates a T cell immune response against cancer cells (+ for PAP)	Phases I-II: [61] Phase II: [63–65]

Ipilimumab (Yervoy)	IgG1 Human monoclonal antibody	Blocks the activity of CTLA-4 and Treg expression	Phases I-II: [67] Phase III: [68]

Tasquinimod	Oral quinolone-3-carboxamide	Antitumor action through inhibition of angiogenesis and immunomodulation	Phase III: [69, 70]

Prostvac-VF	Vector based vaccing	A combination of two viral particles, vaccinia, and fowlpox that infect the APC cells promoting an immune response against PSA expressing cells	Phase II: [71]

GVAX	Granulocyte-macrophage colony-stimulating factor (GM-CSF) gene-transfected tumor cell vaccine	Evocation of a strong immunoreaction by antigens expressed on human prostate cell lines modified by GM-CSF	Phase III: [72, 73]
